# International comparison of pressure ulcer measures in long-term care facilities: Assessing the methodological robustness of 4 approaches to point prevalence measurement

**DOI:** 10.1016/j.jtv.2021.01.007

**Published:** 2021-01-24

**Authors:** Mircha Poldrugovac, Michael Padget, Lisette Schoonhoven, Nicola D. Thompson, Niek S. Klazinga, Dionne S. Kringos

**Affiliations:** a Amsterdam UMC, Department of Public and Occupational Health, University of Amsterdam, Amsterdam Public Health Research Institute, Meibergdreef 9, 1105AZ, Amsterdam, the Netherlands; b Independent Research Consultant Paris, France; c Julius Center for Health Sciences and Primary Care, University Medical Center Utrecht, Universiteitsweg 100, 3584 CG, Utrecht, the Netherlands; d School of Health Sciences, Faculty of Environmental and Life Sciences, University of Southampton, University Road, Southampton, SO17 1BJ, United Kingdom; e Division of Healthcare Quality Promotion, Centers for Disease Control and Prevention, 1600 Clifton Rd, Atlanta, GA, 30333, USA

**Keywords:** Benchmarking, Long term care, Nursing homes, Pressure ulcer, Quality indicators, Health care, Quality of health care

## Abstract

**Introduction:**

Pressure ulcer indicators are among the most frequently used performance measures in long-term care settings. However, measurement systems vary and there is limited knowledge about the international comparability of different measurement systems. The aim of this analysis was to identify possible avenues for international comparisons of data on pressure ulcer prevalence among residents of long-term care facilities.

**Material and methods:**

A descriptive analysis of the four point prevalence measurement systems programs used in 28 countries on three continents was performed. The criteria for the description and analysis were based on the scientific literature on criteria for indicator selection, on issues in international comparisons of data and on specific challenges of pressure ulcer measurements.

**Results:**

The four measurement systems use a prevalence measure based on very similar numerator and denominator definitions. All four measurement systems also collect data on patient mobility. They differ in the pressure ulcer classifications used and the requirements for a head-to-toe resident examination. The regional or country representativeness of long-term care facilities also varies among the four measurement systems.

**Conclusions:**

Methodological differences among the point prevalence measurement systems are an important barrier to reliable comparisons of pressure ulcer prevalence data. The alignment of the methodologies may be improved by implementing changes to the study protocols, such as aligning the classification of pressure ulcers and requirements for a head-to-toe resident skin assessment. The effort required for each change varies. All these elements need to be considered by any initiative to facilitate international comparison and learning.

## 1. Introduction

Performance measurement in long-term care is a challenge of growing importance as the population ages and the demand for long-term care services increases [1]. There is increased interest in international benchmarking of the quality of long-term care services provided in different healthcare systems, as it has considerable potential to improve patient outcomes [2]. International benchmarking is essential to signal differences between countries and can enhance learning across countries by exploring the reasons behind the differences. International benchmarking has been used effectively in other areas, such as acute care [3]. Some proposals for indicators in long-term care with potential for international comparisons have been published recently [2,4]. However, they require considerable data collection capabilities, which many countries do not have at the moment [5].

An alternative approach for comparisons of performance of long-term care facilities across countries is to take advantage of existing performance measurement initiatives. In such an approach the central question is whether the data being collected by different performance measurement initiatives produce comparable rates. The Organization for Economic Cooperation and Development (OECD), an international organization of mostly high- and middle-income countries, recently used such an approach to publish data on pressure ulcer prevalence in long-term care facilities [3].

Comparing indicator rates, collected through different performance measurement initiatives, is generally undesirable, as actual differences in performance are likely to be skewed by differences in measurement methodologies [6]. At the same time, the establishment of internationally coordinated multi-country performance measurement initiatives with a broad inclusion of countries is difficult to achieve [7]. Starting with existing performance measurement initiatives and modifying them if necessary to enhance comparability, might have a higher likelihood of success.

Pressure ulcers in long-term care facilities are a strong candidate for internationally comparable measurements. Pressure ulcer indicators are among the most frequently used measures of the quality of care provided in long-term care settings [8,9]. A high level of international standardization of definitions of pressure ulcers has been achieved based on expert consensus [10]. This suggests an increased likelihood that independent pressure ulcer measurement systems could produce comparable results. Considering the existing wide use of pressure ulcer indicators and the relative homogeneity of measurement, pressure ulcers indicators have been chosen as the focus of our analysis.

In order to calculate performance indicators, such as pressure ulcer rates, long-term care facilities’ resident level data may be collected via different approaches. Two approaches prevail in high- and middle-income countries: one involves a cross-sectional ad hoc survey that is repeated periodically and produces point prevalence measures. The other approach involves using data collected continuously by long-term care facilities to monitor the wellbeing of residents. A notable example of the latter approach is the Resident Assessment Instrument/Minimum Data Set [5,11,12]. Currently this instrument is only used in a few countries [5] and an international common minimum dataset in long-term care is still not widely established [13]. Hence the most promising approach to obtaining internationally comparable performance data in long-term care in the short run is based on point prevalence survey initiatives, on which our study focuses.

We identified four point prevalence survey programs which include data on pressure ulcer prevalence in long-term care facilities (Box 1) from 28 high- and middle-income countries spanning three continents: the Healthcare-Associated Infections in Long-Term Care Facilities (HALT) survey, coordinated by the European Centre for Disease Prevention and Control (ECDC) [14], the Nursing Home Prevalence Survey undertaken within the Centers for Disease Control and Prevention (CDC) Emerging Infections Program (EIP) in the United States [15], the point prevalence survey performed within the Pressure Injury Prevention Project (PIPP), coordinated by the Clinical Excellence Commission in New South Wales, Australia [16], and the Landelijke Prevalentiemeting Zorgproblemen (LPZ) survey, coordinated by the Living Lab in Ageing and Long-term Care of Maastricht University in the Netherlands [17].

This paper aims to identify possible avenues for international comparisons of data on pressure ulcer prevalence among residents of long-term care facilities by comparing and analyzing these four point prevalence measurement systems. More specifically, this study aims to answer the questions:

- What are the key methodological features of the four point prevalence measurement systems?
- To what extent do these measurement systems have the ability to adhere to a common set of methodological criteria to facilitate international comparisons of the prevalence of pressure ulcers in long-term care facilities?

## 2. Materials and methods

### 2.1. Choice of pressure ulcer measurement systems

The four point prevalence measurement systems analyzed represent a convenience sample of point prevalence measurement initiatives of pressure ulcers in long-term care facilities. The specific choice of these four systems was guided by a number of considerations. All four systems have been established as a program to be repeated over time, as opposed to being a one-time evaluations. The programmatic nature of the measurement systems is important to confirm that they can be sustained in time and hence could be used as a standing instrument allowing international comparisons. Two of the initiatives (EIP and PIPP) are limited to single countries, while the other two are multi-country initiatives. The implication is that the assessment will include comparisons of subnational, national, and multi-country initiatives. The geographic spread of the included initiatives means that all three regions and countries (Europe, United States, and the Pacific area) represented by the organizations that published the international guideline on pressure ulcers in 2019 [10] are included in the analysis.

### 2.2. Analysis of the measurement systems

A descriptive analysis was performed to outline key features of the point prevalence measurement systems. The description follows a list of items developed by reviewing the literature. In particular, we considered published criteria for indicator selection [6,23,24], important methodological features for international comparisons [25,26] and challenges specific to pressure ulcer measurement [27–30].

The list of items used to describe the point prevalence measurement systems are grouped around three high level issues: the breadth of the measurement system, its accuracy, and the structures in place to support the data collection process. These issues and the items within them have been selected taking into account the specific purpose of this analysis, i.e. using existing measurement systems for international comparisons. Items describing the breadth of the four initiatives were considered important to give a sense of their scope and likely scalability. Items related to the accuracy of the four point prevalence systems inform the expected validity and reliability of the measurements. The items used to describe the supporting structures are potential indicators of the ease with which features of data collection methodologies of each point prevalence system can be modified. Such modifications may be necessary to adapt to an internationally agreed common set of methodological criteria.

In addition to describing the key features of the four point prevalence survey programs, we also considered whether the pressure ulcer prevalence rates obtained from these surveys could produce comparable rates. To compare results from different point prevalence surveys, they must share key methodological specifications. These specifications do not necessarily signal a methodology of superior or inferior quality, but need to be consistent among the surveys to be able to provide comparable results. The set of reference specifications against which the comparability of the surveys was assessed was based on an iterative process of reviewing the methods used by the four point prevalence surveys. This set of specifications focused on 3 fundamental elements of an indicator: the numerator, the denominator and items related to stratification. For each of these elements key items that point to comparability were described.

In the case of pressure ulcer definition, the classification presented in the international guideline co-published by the National Pressure Injury Advisory Panel, the European Pressure Ulcer Advisory Board and the Pan Pacific Pressure Injury Alliance is used for our study. Information on the definitions and methods used for each of the four point prevalence surveys was retrieved from published documents and information provided directly by the investigators involved in some of these point prevalence systems.

## 3. Results

### 3.1. Relevant key features of the four point prevalence measurement systems

The key features of the point prevalence surveys are presented in Table 1. The breadth of the four surveys varies substantially. A major difference between the point prevalence systems is their purpose. The HALT and EIP surveys are focused on healthcare-associated infections and antimicrobial use, and pressure ulcer data are collected as a relevant risk factor. This is not the case for the PIPP and LPZ surveys, which put explicit emphasis on pressure ulcer data collection. All of the surveys are part of a broader quality improvement effort, where facility level data are intended for internal use by the surveyed facility and aggregated data are intended for the general public or policy-makers.

The PIPP and LPZ surveys, where pressure ulcer measurement is one of the principal aims, collected more detailed data on pressure ulcers, such as category (i.e., stage or grade) of the pressure ulcer or their location and presence on admission. Furthermore, these two measurement systems also require direct patient assessment for pressure ulcers by surveyors, while the HALT protocol does not explicitly require such an examination and the EIP survey is based on existing documentation in resident medical records. These findings suggest that the accuracy of the PIPP and LPZ surveys in measuring pressure ulcers is likely higher than the other two point prevalence systems. All four point prevalence surveys took action to train the surveyors and increase the validity and reliability of the surveys. However, the training and reliability and validity efforts were related to the survey methods and hence in the case of HALT and EIP were not focused on pressure ulcer measurement.

All four point prevalence systems use an ad hoc software or data collection interface. All of these four systems also have a two tier coordination arrangement, where in addition to the overall coordinating institution, there are local entities to streamline communication between long-term care facilities and the overall coordinating body. These local entities are national survey coordinators and national project groups in the case of HALT and LPZ, respectively, state public health authorities in the case of EIP, and so-called Local Health Districts and Specialty Health Networks in the case of PIPP.

### 3.2. Methodological comparability of the measurement systems

The ability of the four point prevalence measurement systems to adhere to common methodological specifications is presented in Table 2. Only the LPZ and PIPP point prevalence measurement systems make an explicit reference to the international guidelines [10,17,21]. The HALT protocol requires the inclusion of all pressure ulcer categories, however there is no explicit reference to deep tissue injury or unstageable pressure ulcers [14]. The CDC survey includes the four pressure ulcer categories and unstageable pressure ulcers, but no explicit reference is made to deep tissue injury. At the same time all of the four point prevalence systems gather data on the number of patients with pressure ulcers (as opposed to the total number of pressure ulcers, for example) and include category 1 pressure ulcers.

A common definition for long-term care facilities was formulated as reported in Table 2. Nonetheless, this does not imply that the long-term care facilities included by the various point prevalence measurement systems are the same. The HALT survey for example identified five types of long-term care facilities (General nursing homes, Residential homes, Specialized long-term care facilities, Mixed long-term care facilities and Other long-term care facilities) [14], all of which can fit the general definition used in this analysis. Within the sample of eligible long-term care facilities in each point prevalence measurement system, the LPZ survey does not include a random sample of facilities, as participation to the survey is voluntary. The PIPP survey includes all of the long-term care facilities under the state authority, but these represent only part of the long-term care facilities in the state, as most fall under the authority of the central Australian Government. The EIP survey is based on a random sample of long-term care facilities, but participation is voluntary. The HALT protocol does recommend a random choice of long-term care facilities in each country, but not all participating countries are able to satisfy this requirement. The HALT survey also set out criteria to assess national representativeness of the sample of long-term care facilities [14]. All four point prevalence measurement systems require that all residents within the selected long-term care facility, department or unit are assessed.

Crude data are provided to the coordinating organization within all four point prevalence measurement systems. All four systems also collect information on impaired mobility, although the way they define it differs slightly. For example the HALT and EIP surveys collect data on “residents who need a wheel chair or are bedridden on the PPS [Point prevalence survey] day” [14] while the LPZ systems collect data on mobility on a 5 point scale based on the Care Dependency Scale [17].

## 4. Discussion

The four point prevalence measurement systems differ considerably on a number of features. The ability to correctly identify and classify pressure ulcers requires a physical assessment of the resident. The international guideline [10] explicitly recommends a head-to-toe skin assessment. The PIPP and LPZ surveys do require a skin assessment of the resident. In the case of the EIP survey this is not performed by surveillance officers collecting the prevalence survey data. However, the surveillance officers have access to nursing home documentation that has been shown to be highly reliable in pressure ulcer identification [42]. The HALT survey requires a review of the residents with health personnel, but not necessarily a skin assessment of the patient.

The ability to accurately distinguish patients who have a pressure ulcer from those who do not have one also relies on the case definition of pressure ulcer and the classification related to it [30,43]. Despite the availability of an international guideline on pressure ulcer definition and classifications [10], two of the four measurement systems analyzed do not make an explicit reference to it. Their definitions of what is considered a pressure ulcer do not contradict the international guideline definition, but also do not clarify whether suspected deep tissue injury should be included in the count or not.

The lack of explicit guidelines on deep tissue injury in the HALT and EIP surveys and on unstageable pressure ulcer in the HALT survey may hamper comparability [9,28]. However, it should be noted that these types of pressure ulcers are not very common. On the contrary, category 1 pressure ulcers are the most frequent category present [44,45]. In the latest report of the PIPP survey [21], 64% of all pressure ulcers acquired in the long-term care facility were classified as category 1. A recent systematic review of pressure ulcers in Europe calculated a mean percentage of category 1 pressure ulcer to be 32.35% [46]. Errors in classifying category 1 pressure ulcer may therefore lead to important differences in pressure ulcer prevalence counts. The correct identification of category 1 pressure ulcer was relatively low in several studies [47,48].

The accuracy of pressure ulcer monitoring may be increased by excluding category 1 pressure ulcer from the count. Category 1 pressure ulcer are indeed not included in several pressure ulcer reports [28,33,49]. As practices in excluding category 1 pressure ulcer tend to differ between studies [50,51], both the HALT and the EIP surveys explicitly mentioned that category 1 pressure ulcer (non-blanchable erythema) is to be included in the data collection. It is worth noting that category 1 pressure ulcers are clinically important, despite challenges in accurate measurement [52,53]. If data on pressure ulcer category were collected by all point prevalence measurement systems, it would also be possible to compare data on pressure ulcer prevalence excluding category 1. This might provide an additional piece of information about the comparability of the measurement systems, without loss of important clinical information.

Another crucial aspect for accurate monitoring of pressure ulcers is the ability to correctly identify pressure ulcers. Kottner et al. [43] in their systematic review found high interrater reliabilities of pressure ulcer classification based on skin examination. However, they also found that the studies considered included raters “specialized, trained or experienced in pressure ulcer diagnosis” [43]. On the other hand, when a convenience sample of nurses from five European countries was surveyed, the reliability of their classification of pressure ulcer based on photographs was considerably lower [47]. Training can improve the ability of nurses to correctly identify pressure ulcers [54]. The training reported in the results section above refers to the overall point prevalence measurement systems and is not necessarily limited to training in pressure ulcer identification and classification. In particular the HALT survey may not emphasize the training in pressure ulcer identification and classification, as pressure ulcers are not the main focus of the survey. The same also holds for the EIP survey, but in that case surveillance officers were reliant on the documentation in resident medical records to identify pressure ulcers.

The denominator in all four measurement systems is represented by all residents of the unit of the long-term care facility being surveyed on the day of the survey. However, the long-term care facilities included in the denominator do not necessarily serve residents with similar needs and health status. Several studies have found varying proportions of the older population residing in long-term care facilities and different residents characteristics in different countries [55,56]. These differences are also apparent when the number of long-term care beds relative to the size of the population aged 65 and over in different countries is compared [3]. A concern may arise when comparing long-term care facilities with different purposes or characteristics that potential differences in the susceptibility of the residents (i.e. the population in the denominator) to develop pressure ulcers might limit comparability. Considering the heterogeneity of long-term care facilities described above, the best approach to take into account these differences is to include information on risk factors for pressure ulcer development.

There are several risk factors for pressure ulcer development [10]. Those that emerge most frequently as independent risk factors have been grouped by Coleman et al. [57] in three domains: mobility/activity, perfusion (including diabetes) and skin/pressure ulcer status. While all four point prevalence measurement systems analyzed collect data on mobility, the way that mobility was defined differs; all four can identify residents with severe mobility limitations for the purpose of comparability. Several tools exist to assess the risk of developing a pressure ulcer [58,59] which, if integrated into the measurement systems, could be used to provide a measure for the susceptibility of residents to develop pressure ulcers. These tools, however, require additional data which are not currently collected by all of the four point prevalence measurement systems under consideration.

It is important to note that the HALT survey protocol includes recommendations, such as the minimum number of long-term care facilities per country, to which countries adhere to a varying extent, as difference in representativeness and deviations from recommended country sample sizes indicate [14,34]. While this premise weakens the comparability of the findings, it might also be necessary, to allow enough flexibility for each country to implement the protocol to the extent that the resources and engagement of each country on the issue allow.

A few low resource interventions might improve the quality of the pressure ulcer measurement in particular in the case of the HALT and EIP surveys, which are not focused on pressure ulcers, such as clarifying the definition of pressure ulcer and collecting data on the category of pressure ulcer identified. The quality of the collected data may be increased by ensuring that a resident head-to-toe assessment is the basis of pressure ulcer data collection. This would also imply the need for the point prevalence measurement system training to include pressure ulcer recognition and classification. However, such an intervention is resource intensive, may have legal, ethical and other implications in some countries. Alternatively, assurances may be sought of the reliability of existing pressure ulcer data that surveyors could use.

It is also important to consider the intended use of the performance measures. Small variations in quality of data collection may not be acceptable for a nursing home fine-tuning its pressure ulcer prevention activities, but might be acceptable for country comparisons, where the purpose of such comparison is for countries to recognize whether they are outliers on pressure ulcer prevalence with respect to their peers. Such outliers may find that they need to pay more attention to the issue of pressure ulcer in long-term care facilities nationwide. A recent publication of the OECD based on pressure ulcer data from the HALT and EIP surveys showed a 14-fold difference in prevalence rates between the best and worst performers [3]. Caution is necessary in interpreting these differences. The precision of prevalence rates is impacted by factors such as the number of participating institutions and included residents. Our analysis suggests that other factors, such as representativeness of the sample and approach to data collection, are also important.

The LPZ and the PIPP surveys use relatively resource intensive pressure ulcer measurement methods, which provide data of good quality but present the challenge of scalability. The PIPP survey is limited to a group of long-term care facilities in one Australian state. The LPZ survey, which has an international breadth, involves voluntary participation of long-term care facilities. The latter can be assumed to have a high level of commitment to the issue of pressure ulcer, which limits country representativeness of the findings. The HALT and EIP surveys have some limitations in the quality of the measurement systems as explained above, which are likely related to the fact that the point prevalence measurement systems are not focused on pressure ulcer measurement. If the pressure ulcer identification accuracy and sample representativeness limitations could be overcome, the pressure ulcer values provided by the four point prevalence measurement systems would be comparable for the purpose of steering policies at national level. All recommendations to improve international comparability of pressure ulcer rates are summarized in Box 2.

### 4.1. Strength and limitations

Our analysis includes point prevalence measurement systems used in 28 high- and middle-income countries on three continents, thus providing a strong international perspective.

We focused specifically on four point prevalence measurement systems; additional observations might be drawn from analyzing other point prevalence measurement systems aimed at monitoring pressure ulcers. There are also other measures of pressure ulcers, in particular incidence measures, which can be derived from long-term care facility-based surveillance systems. A separate but equally important line of investigation would be to assess the quality, international comparability and availability of measures obtained from such data collection systems.

## 5. Conclusions

The four point prevalence measurement systems analyzed vary in some of their key features. They use different classifications of pressure ulcers and different approaches to data collection. The methods to select and include long-term care facilities by country also differs among the measurement systems.

In principle it is possible to harmonize the approaches of these measurement systems. The comparability of the pressure ulcer count depends partly on using a compatible pressure ulcer classification system and either a head-to-toe skin assessment, supported by ad hoc training or validated pre-existing documentation. If inferences are to be made about pressure ulcer prevalence in long-term care facilities by country, then the country representativeness of a comparable long-term care facility sample is another crucial element. Some of the adaptations necessary to increase comparability can be implemented through minor changes in the survey protocols. Other adaptations are more resource intensive and may be less feasible in certain countries. Coordinating bodies of these point prevalence measurement systems should consider the risk and benefits of adapting their systems to enhance international comparability of pressure ulcers measures in long-term care facilities. The comparability of the measures will support learning across countries with the aim to facilitate improvements in pressure ulcer prevention and treatment and ultimately result in increased resident safety.

## Figures and Tables

**Table 1:** Key features of the pressure ulcer measurement systems.

Name of the Measurement system	The Healthcare-Associated Infections in Long-Term Care Facilities survey (HALT) coordinated by the European Centre for Disease Prevention and Control (ECDC)	Emerging Infections Program (EIP) Nursing Home Prevalence Survey coordinated by the Centers for Disease Control and Prevention (CDC), United States	Point prevalence survey performed within the Pressure Injury Prevention Project (PIPP), coordinated by the Clinical Excellence Commission in New South Wales, Australia	Landelijke Prevalentiemeting Zorgproblemen (LPZ) survey, coordinated by Living Lab in Ageing and Long-term Care of Maastricht University, The Netherlands
				
Feature	HALT	EIP	PIPP	LPZ

**Breadth of the measurement system**				
**Primary objective of the data collection system**	Estimation and monitoring of healthcare-associated infections and antimicrobial use in longterm care facilities [14].	Measurement of the prevalence and description of the types of healthcare-associated infections and antimicrobial use in nursing homes.	Measurement of pressure ulcers.	Measurement of the prevalence, preventive measures and treatment of pressure ulcers, malnutrition, falls, restraints, incontinence and pain [17].
**Embedding in a larger quality improvement or accountability system**	Pressure ulcer data are collected as a risk factor for healthcare associated infections. The overall aim is to identify priorities for national and local interventions and evaluate their implementation in participating countries and long-term care facilities [14].	This is an independent public health healthcare-associated infections and antimicrobial use surveillance activity. Pressure ulcer data are collected as a risk factor for healthcare- associated infections and antimicrobial use.	The annual point prevalence survey is part of a broader pressure ulcer prevention initiative, aiming to reduce the occurrence of pressure ulcers and if they do occur, to help reduce the recovery time for the patient [20].	The primary purpose of the project is to give health care organizations insight into their basic care quality. The project also provides information about the prevention and treatment of care problems (22).
Feature	HALT	EIP	PIPP	LPZ
**Time dimension and form of presentation of the results to the target public**	There is an ad-hoc software that can generate preliminary summary reports immediately. ECDC provided facility-level feedback reports (through national coordinators), with comparisons within long-term care facility types when possible. National coordinators may also receive feedback information and may engage in further dissemination activities. The ECDC provides an aggregated (European) report for the whole project, which is in the public domain [14]	Staff at each of the 10 participating states has immediate access to the data for the nursing homes participating from their state. Upon data entry, CDC has immediate access to the data from all nursing homes participating. CDC provided state-level summary reports of preliminary data and EIP staff provided a nursing home feedback report to each participating nursing home within 3–4 months of completing data collection. The EIP surveillance team visited or followed up via phone call or email with all nursing homes to review the survey report and results. CDC has led development of abstracts for presentation at scientific meetings and manuscripts for publication in peer-reviewed journals – ongoing [31,32]	The Clinical Excellence Commission developed the Quality Audit Reporting System, through which health entities collect data privately. Access to the system is not open to the general public. Ward-level data are available to health entities for improvement. Aggregate data at state-level are public. Additionally, there is a public report published each year for the previous year.	Each participating institution has access to its own results and national data for benchmark purposes. The data are presented in specially designed digital dashboards with interactive features. The National project groups receive a full data file for research and analysis purposes [17].
**Level of reporting (to the coordinating body)**	Coordinating institution (ECDC) receives data at long-term care facility-level. The collection of individual and ward-level data is recommended but not required for internal use [14].	CDC, the coordinating institution, receives data at the nursing home resident-level. Data can be aggregated at the location (ward/unit), nursing home facility, state, and overall levels.	Reporting to the coordinating body via Quality Audit Reporting System tool is at ward-level.	National/Ward/department-level data are available to participating institutions [17,33].
**Number of countries included in the latest data collection**	25 European Countries [34]	1 country (United States); 10 states that participate in the CDC Emerging Infections Program [35]	1 state in Australia (New South Wales, Australia)	4 Countries (The Netherlands, UK, Turkey and Austria). In Switzerland only hospitals participate in the project
**Total number of longterm care facilities included in the most recent data collection**	1788 [34]	161 nursing homes from within the 10 states (above).	67 NSW Health Residential Aged Care facilities, 97% of all facilities under the responsibility of state authorities [21]	509
**Total number of residents included in the most recent data collection**	102,301 [34]	15,276 residents within the participating nursing homes on the day the prevalence survey was conducted.	981 or 92% of residents [21]	42,000
**Accuracy of the measurement system**				
**Pressure ulcer information collected (category, location, number, present on admission)**	Data on the number of patients with a pressure ulcer is collected. The data does not include information about category, number of pressure ulcers in a single patient, location or presence on admission [14].	Data on the presence or absence of a pressure ulcer (of any category or unstageable) on the prevalence survey date for every resident in participating nursing homes is collected. The pressure ulcer category, location number or presence on admission is not collected.	Data collected include category (all 6 categories) and location for up to 4 (of the most severe) pressure injuries. Present on admission data is also collected [16].	Information on category, location, number and presence on admission are collected.
**Coverage/sampling**	All eligible residents should be included in the survey. Eligibility includes living full time in the long-term care facility, being present at 8:00 a.m. on the day of the survey (which excludes hospitalized patients, for example) and not being discharged from the long-term care facility at the time of the survey [14].	Residents present in the facility at 8:00 a.m. on the survey date and the day before. If a resident was away from the facility for a few hours for a medical appointment (for example, attending a doctor or dental appointment, or receiving outpatient dialysis treatment), or for social or recreational purposes (for lunch or to go shopping) then they are an eligible resident. If the resident has an admission date that is either the same day as the survey date or the day before the assessment date, they are not eligible for inclusion.	It is recommended that at least 85% of people on the unit/ward/ service on the day of the survey have the complete survey attended (this includes the visual skin inspection). 2018 results show that 97% of residential aged care facilities undertook the survey in 2018 and that it included 92% of residents [21].	Participation of both organizations and clients is voluntarily. All eligible residents should be included in the survey. Residents are eligible if they are present at the long-term care facility on the day of the survey and they or their legal representative has given informed consent. Only wards or departments with a response of at least 90% are included in the national calculations [17].
**Training or instructions provided to auditors/surveyors**	With respect to the latest iteration of the survey (called HALT 3) a train-the-trainer workshop was organized in Stockholm in 2015. Training material was provided with a recommendation to regional/national coordinators to organize at least one one-day information and training session (in relation to the whole HALT study and not just the pressure ulcer measurement) [14].	In-person 2-day training for EIP surveillance officers who were engaged in prevalence survey data collection, held at CDC in Atlanta, Georgia in March 2017. Use of standardized data collection form instructions by data collectors. Bi-monthly teleconference calls with data collectors to answer questions and discuss complicated or confusing resident/data collection scenarios.	Surveyors are trained to ensure inter-rater reliability and given instruction in completing the required documentation. They are required to complete the Health Education & Training Institute (HETI) Pressure Injury Prevention modules [16].	Coordinators at each of the participating institutions are trained by the national research group in each country on how to manage the measurement and how to use the questionnaire and the web-based data-entry program. Additionally institutional coordinators receive the study protocol and training package with questionnaire manual and guidelines [17].
**External expert participation to patient examination**	Patient examination is not required to assess pressure ulcers. Depending on available resources, the protocol foresees either local data collectors or local data collectors supported by an external data collector [14].	No direct resident examination was performed for data collection. All data were collected from retrospective review of documentation within each participating nursing home by trained surveillance officers in each state. Documentation in nursing home records on pressure ulcers would typically be performed by nursing home attending physicians, nurses, or specialized wound care nurses.	It is required that at least one member of the survey team is not from the unit being surveyed [16].	Each patient is assessed by two healthcare professionals, one is from the department or ward where the resident is located, while the other is from another ward or department [17].
**Validity testing information**	The study protocol built on previous projects, such as the Improving Patient Safety in Europe project. The working group based at ECDC also sought further inputs on the protocol from European Union experts. The protocol was then piloted in several countries [36,37]. The protocol for a validation survey to take place in some case, synchronous with the original data collection has been developed [38]	A pilot prevalence survey to test data collection forms, data collection instructions, and data collection procedures was conducted in 9 nursing homes in 4 states. The pilot informed definitions, instructions, and data collection practices for the larger prevalence survey performed in 2017 [32].	The survey was piloted prior to state-wide rollout.	The LPZ instrument is based on several previously tested instruments (BRADEN, MUST, CDS). Broad consultation with Dutch and international experts ensured face validity [17].
**Reliability testing information**	The validation surveys (above) also allow reliability check. However those reliability checks were not required to include pressure ulcer data [38].	Due to concerns about validity and reliability of the data collection when performed by nursing home staff (based on pilot findings, above), all data collection was performed by trained surveillance officers from the 10 states of the CDCs Emerging Infections Program (see above). Database system checks at the time of data entry were employed to prevent erroneous, illogical and missing data entry. After data entry, CDC sent a data cleaning report to surveillance officers at each state listing outlier, unusual, and potentially illogical values for their reviewed and verification or correction.	Two survey staff performing the skin assessment (simultaneously) should agree on lesion type and category [39]	Assessments are performed by 2 professionals. Interrater reliability (for hospitals, nursing homes and home care) was found to be good. When necessary, documents were translated and back-translated [17].
**Frequency of the measurement**	Approximately every 3 years, since 2010. The latest survey was undertaken in 2016 and 2017 [14].	To be determined. The first prevalence survey in nursing homes was performed in 2017.	Annually between 2015 and 2018 [21]	Each participating institution can opt to perform the point prevalence survey twice a year. Most long-term care facilities have decided to perform the survey annually.
**Supporting structures Information infrastructure supporting data collection**	A stand-alone software has been developed for this project. It is available to long-term care facilities and national coordinators. The software stores data locally and therefore the data need to be exported to the coordinating organization (ECDC) [14].	A custom, secure internet-based (.NET) database was developed for this project. Data collected from nursing homes in all 10 states is available to and can be exported for analysis by CDC. States have access to and can export the data for nursing homes within their state. Data entry is performed by EIP surveillance officers for the nursing homes within their state. Participating nursing homes do not have direct access to the data entry system, but can request an export of their data at any time.	Reporting on pressure ulcer within the point prevalence survey is integrated in the Quality Audit Reporting System.	A specially designed web-based data-entry program is used to exchange information between participating institutions and coordinating organizations [17].
**Coordination centre of the measurement system**	The ECDC coordinates the whole study. National survey coordinators are identified to streamline communication with long-term care facilities and coordinate activities nationally [14].	CDC coordinates the prevalence survey design and data collection. The 10 states coordinate nursing home recruitment, data collection, and data entry within their respective states.	The coordinating institution is the Clinical Excellence Commission, which supports Local Health Districts (LHDs) and Specialty Health Networks (SHNs).	The LPZ project group at the Living Lab in Ageing and Long-term Care (Maastricht University) coordinates the whole project. Additionally each country has a national project group [17].

**Table 2:** Ability of point prevalence pressure ulcer measurement system to adhere to a set of common methodological specifications.

Name of the Measurement system	The Healthcare-Associated Infections in Long-Term Care Facilities survey (HALT) coordinated by the European Centre for Disease Prevention and Control (ECDC)	Emerging Infections Program (EIP) Nursing Home Prevalence Survey coordinated by the Centers for Disease Control and Prevention (CDC), United States	Point prevalence survey performed within the Pressure Injury Prevention Project (PIPP), coordinated by the Clinical Excellence Commission (CEC) in New South Wales, Australia	Landelijke Prevalentiemeting Zorgproblemen (LPZ) survey, coordinated by Living Lab in Ageing and Long-term Care of Maastricht University (MU), The Netherlands
				
Methodological specifications	HALT	EIP	PIPP	LPZ

**Numerator-related items**				
A pressure ulcer is defined according the international guideline co-published by the National Pressure Injury Advisory Panel, from the United States, the European Pressure Ulcer Advisory Board and the Pan Pacific Pressure Injury Alliance [10,40]	No. “All grades of pressure sores should be included (e.g. the lowest grade, non-blanching erythema, characterized by discoloration of intact skin not affected by light finger pressure)” [14]	No. Defined as - pressure ulcers in all locations on the body and of all categories (1 through 4, and unstageable), even category 1 characterized by discoloration of intact skin (non-blanching erythema)	Yes	Yes
Numerator is number of patients with a pressure ulcer	Yes	Yes	Yes	Yes
All pressure ulcer categories are included in the count	Yes	Yes	Yes	Yes
Information on pressure ulcers was obtained via ad hoc patient examination by a trained nurse	No	No	Yes	Yes
**Denominator related items**				
Long-term care facilities included in the point prevalence survey can fit the definition of organizations that provide a variety of services, both medical and personal care, to people who are unable to live independently [41].	Yes	Yes	Yes	Yes
All long-term care facilities in the country/region or random sample of all long-term care facilities are included	Yes, this is the recommendation. However, it is not achieved by all participating countries.	Random sample of nursing homes; voluntary participation.	All residential aged care under state authority.	No
All residents or random sample of all residents are included in the denominator (not only those at risk)	Yes	Yes	Yes	Yes
**Items related to stratification**				
Crude data on numerator and denominator are collected by coordinating organization	Yes	Yes	Yes	Yes
Data on the number of patients with low mobility is available	Yes	Yes	Yes	Yes

**Box 1:** Short description of the context of four point prevalence survey programs of long-term care facilities which include pressure ulcer measurements.

The Healthcare-Associated Infections in Long-Term Care Facilities survey (HALT)	Emerging Infections Program (EIP) Nursing Home Prevalence Survey
*Coordinated by the **European Centre for Disease Prevention and Control (ECDC)*** *Coverage: **Europe*** HALT is a point prevalence survey that focuses on healthcare associated infections and antimicrobial use. The first survey in long-term care was performed in 2010 and was based on a previous feasibility study and the work done within the Healthcare-Associated Infections Surveillance Network within the ECDC [18].	*Coordinated by **Centers for Disease Control and Prevention (CDC)*** *Coverage: **United States*** “The EIP network is a national resource for surveillance, prevention, and the control of emerging infectious diseases“ established in 1995 [19]. Through this network the first nursing home prevalence survey focused on healthcare associated infections and antimicrobial use was conducted in 2017 (15).
Pressure Injury Prevention Project (PIPP)	The Landelijke Prevalentiemeting Zorgproblemen (LPZ)
*Coordinated by **Clinical Excellence Commission*** *Coverage: **New South Wales, Australia*** The project was established in 2012 to support pressure ulcer prevention and management [20]. Within the project a point prevalence survey of long-term care facilities focused on pressure injuries management has been performed annually since 2015 (21).	*Coordinated by **Living Lab in Ageing and Long-term Care of Maastricht University*** *Coverage: **The Netherlands, UK, Turkey and Austria*** The project is a point prevalence measurement of the quality of care in long-term care facilities, which originated in the Netherlands. It began in 1998 with the measurement of pressure ulcer management and since 2004 includes other aspects of quality care: incontinence, malnutrition, use of restraints, falls and pain [22].

**Box 2:** Recommendations to improve international comparability of pressure ulcer measures in long-term care facilities

• Data collection should include specification of pressure ulcer category.
• Guidelines in point prevalence system protocols should specify how to count unstageable pressure ulcers and deep tissue injuries.
• In all cases, where an underlying pressure ulcer data collection system has not been validated, ad-hoc head-to-toe resident assessment by trained professionals should be required as part of the point prevalence survey.
• Country comparisons of pressure ulcer rates require representative samples of institutions and their residents, with sample sizes depending the desired accuracy.

## Abbreviations:

ECDC: the European Centre for Disease Prevention and Control
HALT: Healthcare-Associated Infections in Long-Term Care Facilities survey
CDC: Centers for Disease Control and Prevention
EIP: the Nursing Home Prevalence Survey undertaken within the CDC Emerging Infections Program
PIPP: the point prevalence survey performed within the Pressure Injury Prevention Project
LPZ: Landelijke Prevalentiemeting Zorgproblemen survey
